# Head–Eye Vestibular Motion Therapy Affects the Mental and Physical Health of Severe Chronic Postconcussion Patients

**DOI:** 10.3389/fneur.2017.00414

**Published:** 2017-08-22

**Authors:** Frederick Robert Carrick, Joseph F. Clark, Guido Pagnacco, Matthew M. Antonucci, Ahmed Hankir, Rashid Zaman, Elena Oggero

**Affiliations:** ^1^Bedfordshire Centre for Mental Health Research in Association with University of Cambridge, Cambridge, United Kingdom; ^2^Neurology, Carrick Institute, Cape Canaveral, FL, United States; ^3^Harvard Macy Institute and MGH Institute of Health Professions, Boston, MA, United States; ^4^Department of Neurology and Rehabilitation Medicine, University of Cincinnati College of Medicine, Cincinnati, OH, United States; ^5^Electrical and Computer Engineering Department, University of Wyoming, Laramie, WY, United States; ^6^Neurology, Plasticity Brain Center, Orlando, FL, United States; ^7^Psychiatry, Carrick Institute, Cape Canaveral, FL, United States; ^8^Department of Psychiatry, University of Cambridge, Cambridge, United Kingdom

**Keywords:** neuro-otology, head movement, eye movement, vestibular, concussion, mTBI, postconcussion syndrome, C3 Logix

## Abstract

**Context:**

Approximately 1.8–3.6 million annual traumatic brain injuries occur in the United States. An evidence-based treatment for concussions that is reliable and effective has not been available.

**Objective:**

The objective of this study is to test whether head–eye vestibular motion (HEVM) therapy is associated with decreased symptoms and increased function in postconcussive syndrome (PCS) patients that have been severely impaired for greater than 6 months after a mild traumatic brain injury.

**Design:**

Retrospective clinical chart review.

**Setting and participants:**

Tertiary Specialist Brain Rehabilitation Center.

**Interventions:**

All subjects underwent comprehensive neurological examinations including measurement of eye and head movement. The seven modules of the C3 Logix Comprehensive Concussion Management System were used for pre- and postmeasurements of outcome of HEVM therapy.

**Materials and methods:**

We utilized an objective validated measurement of physical and mental health characteristics of our patients before and after a 1-week HEVM rehabilitation program. We included only PCS patients that were disabled from work or school for a period of time exceeding 6 months after suffering a sports concussion. These subjects all were enrolled in a 5-day HEVM rehabilitation program at our Institutional Brain Center with pre- and post-C3 Logix testing outcomes.

**Results:**

There were statistical and substantive significant decreases in PCS symptom severity after treatment and statistical and substantive significant increases in standardized assessment of concussion scores. The outcomes were associated with positive changes in mental and physical health issues. This is a retrospective review and no control group has been included in this study. These are major limitations with retrospective reviews and further investigations with prospective designs including a randomized controlled study are necessary to further our understanding.

**Conclusion:**

Head–eye vestibular motion therapy of 5 days duration is associated with statistical and substantive significant decreases of symptom severity associated with chronic PCS.

## Introduction

Traumatic brain injury (TBI) is caused by extracranial mechanical forces. These injuries may be associated with a loss of consciousness and memory impairment for recent events surrounding the head injury with changes of mental status at the time of the injury (1). Unfortunately, there is a lack of reliable and efficient evidence-based treatments for the approximately 1.8–3.6 million traumatic brain injuries that are reported annually in the United States (2) and we wanted to contribute to better clinical outcomes. Mild traumatic brain injury (mTBI), even in the chronic phase years postinjury, is not a benign condition but is associated with increased rates of headaches, sleep problems, and memory difficulties. Furthermore, mTBI can complicate or prolong recovery from preexisting or comorbid conditions such as post-traumatic stress disorder, a neuropsychiatric condition (3). Such patients may demonstrate difficulty with executive functionality and exhibit a mental inflexibility that may render them powerless to shift their focus between concepts (4).

Alarmingly, 20–30% of patients that suffer a mild closed head injury (mCHI) are affected by the incapacitating syndrome of a postconcussive syndrome (PCS) that complicates recovery and contributes to symptoms that may be considered to be neuropsychiatric in nature (5). In fact, mental health issues are related to a history of concussions resulting in possible severe and long-term influence on PCS patients, families, and friends (6).

The likelihood of depression and PCS increases after mTBI and is linked with reduced psychosocial outcomes including an increased probability of self-reported disability, underemployment, low income, and marital problems (7). Adolescents also may be depressed after suffering a concussion and should be screened for depression (8) due to the psychological sequelae that might impair their psychosocial functioning (9). We are concerned with the adverse long-term psychiatric, neurologic, and psychological morbidities that complicate recovery from PCS. mTBI patients that are depressed report increased mental health issues (10) that may confound diagnostic and therapeutic interventions that might be helpful. Many patients might describe only physical symptoms, and it is important for health care providers that attend PCS patients to consider the mental health of their patients. For example, there is a greater risk of suicide in military veterans that have suffered a TBI than those veterans who have not (11). We understand that depression, anger control issues, impairment of cognition and increased incidence of suicide are recognized as a diagnostic feature of chronic traumatic encephalopathy after concussion (12).

An increase in symptoms after concussion does not appear to be related to abnormal structural MRI and microstructural white matter findings. The significant predictors of PCS at 1 month include preinjury mental health problems and the presence of extracranial bodily injuries rather than structural brain disorders (13, 14). We are faced with a public that is exposed to a variety of information specific to head injury in the media with some accurate and inaccurate reporting that may confound treatment applications. Mental health issues have been reported in the media and have contributed to an increased anxiety by PCS patients and their families (15) who are concerned with long-term deficits in cognition and mental health as a consequence of medical mismanagement of concussions (16). Patients and their relatives want to know how long it takes to recover from a concussion. They desire to know if they might have permanent damage to their brains and long-term PCS patients will commonly question if they will ever recover.

Incapacitating PCS symptoms are reported by a majority of children and adolescents within 5 days after a concussion, but 90% arrive at a state of normality for PCS and neurocognition a month after their injury (17). The physical components of PCS are more easily understood and recognized than the neuropsychiatric components (18) even though we know higher rates of depressive symptoms exist in PCS patients when they are compared to the overall population (19).

We are concerned with the global health of our patients with PCS and recognize an ethical obligation of health care providers to protect the present and future mental and physical well-being of their patients (20). Most health care providers do not use instruments to measure or provide a baseline of mental health (21), perhaps because we expect most patients to return to a reasonable quality of life within 6 months of an mTBI. The long time PCS sufferers typically have had persistent symptoms with modifiable psychological risk factors for 1 month (i.e. distress, traumatic stress, and/or low resilience), and at 6 months, they can expect an increase in PCS, depression, traumatic stress, fatigue, insomnia, and a worsening of their quality of life (22).

We desired to study the physical and mental health of subjects that had severe debilitating PCS of greater than 6 months duration. We also wanted to evaluate the effectiveness of a novel PCS treatment in ameliorating both physical and mental health issues. The majority of our long-term symptomatic PCS patients had been treated with a variety of combinations of rest, rehabilitation, and pharmacy that had not been successful. It is understood that the management of sports concussion patients whose symptoms persist greater than 10 days should include cognitive, vestibular, physical, and psychological therapy (23) and we embrace these recommendations.

We understand that the functional integrity of the brain is closely related to eye movement function and that function is compromised postacutely in mCHI especially for saccades, antisaccades, smooth pursuit, and memory-guided sequences (5). We have observed similar cervical–vestibular–ocular pathology in our chronic PCS patients and have developed novel head–eye vestibular motion (HEVM) strategies that have been successful in patient outcomes (24–27). We wanted to measure the consequence of our treatment on both the physical and mental health functions of our sports PCS population. We needed powerful instruments that would provide us with validated outcomes of measurement of our PCS patient’s status and function before and after our therapy. We had experience using the C3 Logix integrated concussion management system developed at the Cleveland Clinic (28) and had found it to be ideal for our patient’s needs. The C3 Logix platform also collects data on the mental health status of PCS patients and is ideal to ascertain whether an HEVM physical rehabilitation modality might be associated with changes in mental health characteristics of PCS patients.

## Materials and Methods

This study was a single-center, retrospective review of records performed at our Institutional Brain Injury Clinic conducted in accordance with the Declaration of Helsinki with equipoise. The records review was approved by the Carrick Institute Institutional Review Board (HHS #: IRB00006615 FWA: 00022305), and written informed consent was obtained from each patient prior to his or her examination and treatment. We identified PCS patients that were disabled from work or school for a period of time exceeding 6 months after suffering a sports concussion. These subjects all were enrolled in a 5-day HEVM rehabilitation program at our Institutional Brain Center with pre- and post-C3 Logix testing outcomes. The review was done by blinded investigators that were not involved in the treatment of subjects nor had any interaction with them or the treating physicians. The C3 Logix integrated concussion management system (28) was used before and after a 1-week HEVM rehabilitation program.

All subjects had their eye and head movements analyzed with Micromedical Technologies Visual Eyes (29) video oculography and a multisensory head–eye (JAZZ-nova) measurement system (30) during the tracking of a sinusoidal smoothly moving visual target in the horizontal and vertical planes. The primary treatment was gaze stabilization exercises administered with coordinated HEVM at positions and speeds associated with a decomposition of head and eye tracking movements. Subjects would attend to a visual target that would move in a plane at a velocity approximating the speed of head–eye decomposition while moving their head in combinations of pitch, yaw, and roll. The visual target underwent a gradual increase of its velocity and amplitude until head–eye movements further degraded or became synchronous at which time the session would stop. These sessions had durations of 3 min at a time followed by a 3-min rest and then repeated three times. The sessions would be scheduled five times per day with a rest period of a minimum of 1.5 h between sessions over 5 days.

Head, eye, and body movements were coordinated by using the Dynavision D2 visual, neurocognitive and rehabilitation system (31). Patients would use coordinated head–eye–body movements to “hit” 64 illuminated random targets encompassing a full visual field. The Dynavision D2 is gamified and trains reaction times and progresses neurocognitive abilities by providing output of reaction and accuracy scores. Patients would train on the Dynavision D2 three times per day for 10 min a session.

A secondary treatment of vestibular and somatic stimulation was administered by placing the patient in an accelerated rotation in a multiaxis rotational chair (MARC) (32) from 0 to 60°/s over 15 s about a plane opposite to the plane of head movements that were slower than coordinated eye movements in combined slow visual pursuits. Subjects underwent 3–30 s acceleration–deceleration rotations with the accelerated rotations beginning at 0 and terminating at 60°/s over 15 s followed by a 15-s deceleration from 60 to 0°/s. The acceleration–deceleration was linear and followed by a 2-min break between each rotation and repeated two times per day over 5 days.

A tertiary treatment of somatic sensory motor movements involved subject complex movements of the upper and lower extremity, both passively with a therapist and actively (right arm, left arm, right leg, and left leg) and in combination (right arm-left leg, left arm-right leg, right arm-right leg, and left arm-left leg). Subjects participated in somatic sensory motor movements for three sessions per day. The eye should not move if the head moves at the same speed of a slow moving target while fovealizing on the target. Neck musculature that exhibits increased tone or resistance to stretch and movement results in a sensory mismatch between head and eye movement. Manipulation of the cervical spine was administered to all patients on the side opposite the greatest eye movement observed with coordinated head eye targeting of slow pursuit targets in the horizontal plane.

### C3 Logix Comprehensive Concussion Management System

C3 Logix consists of seven modules for evaluation, which take approximately 17 min to preform. Four modules are based on long-standing traditional tests that have been translated to electronic form in various incarnations, including the Concussion Symptom Assessment Survey (27 questions on physical condition), standardized assessment of concussion (SAC; including delayed recall), The Trails Test (with and without set switching), and The Processing Speed Task (symbol digit modalities test). The four additional modules include Balance testing (BESS Protocol while capturing accelerometer and gyroscope data and assessing sway volume as well as the standard BESS error score), Simple and Choice Reaction Time and Static and Dynamic Visual Acuity.

Patient symptoms were collected using the C3 Logix graded symptom checklist, derived from 22 standard, publicly accepted symptom survey questions following the recommendation of the 4th International Conference on Concussion in Sport held in Zurich (33). In addition, based on the Cleveland Clinic roll out experience, five extra questions were added to disaggregate the more subtle components of an examinees symptomology (34). The SAC (35, 36) is included in the C3 Logix platform and is derived from existing tests that look at immediate memory, delayed recall, orientation, and concentration.

### Statistical Analysis

Statistical analysis was performed with STATA 14, Statacorp LP, College Station, TX, USA. Two sample paired *t* tests with equal variances were calculated for each variable independent of other variables. The effect size was calculated by Cohen’s *d* to indicate the standardized difference between two means. A Cohen’s *d* of 0.2 is considered to be a small effect size, 0.05 a medium effect size, and 0.08 a large effect size. Multiple regression models of the predictors of severity scores pre- and post-HEVM treatment were calculated as well as the semipartial *R*^2^ of the correlations of symptom severity with each variable to estimate only the unique effect of each predictor in the C3 Logix diagnostic battery. We wanted to know the effect of individual variables as predictors of the symptom severity score and calculated the semipartial *R*^2^ of the correlations of symptom severity with each variable. The semipartial *R*^2^ estimates only the unique effect of each predictor in the C3 Logix diagnostic battery. It is a conservative estimate of the effect of each variable because it measures only how much the *R*^2^ increases when that variable is entered after all the other variables are already in the model controlling for all of the other independent variables.

## Results

We identified 620 subjects suffering from PCS and 70 subjects met the criterion of having persisting debilitating symptoms greater than 6 months, 45 males and 25 females with a mean age of 28 years (SD 8.48). There was a minimum age of 14 and a maximum age of 47. The males had a mean age of 28 years (SD 8.80 minimum age of 14 and a maximum age of 47). The females had a mean age of 29 (SD 8.036 minimum age of 18 and a maximum age of 47). The sports concussions were associated with a variety of activities including ice hockey, lacrosse, American football, soccer, skating, skiing, snowboarding, and gymnastics. A two sample unpaired *t* test with equal variances [*t* (68) = −0.7615, *p* = 0.4490] revealed that there were no statistically significant differences between the symptoms of males and females before HEVM treatment. Post-HEVM treatment demonstrated that there were no statistically significant differences in symptom outcomes between genders after treatment [*t* (68) = −0.0994, *p* = 0.9211]. Therefore, males and females were combined for all statistical analysis.

**Figure 1:**
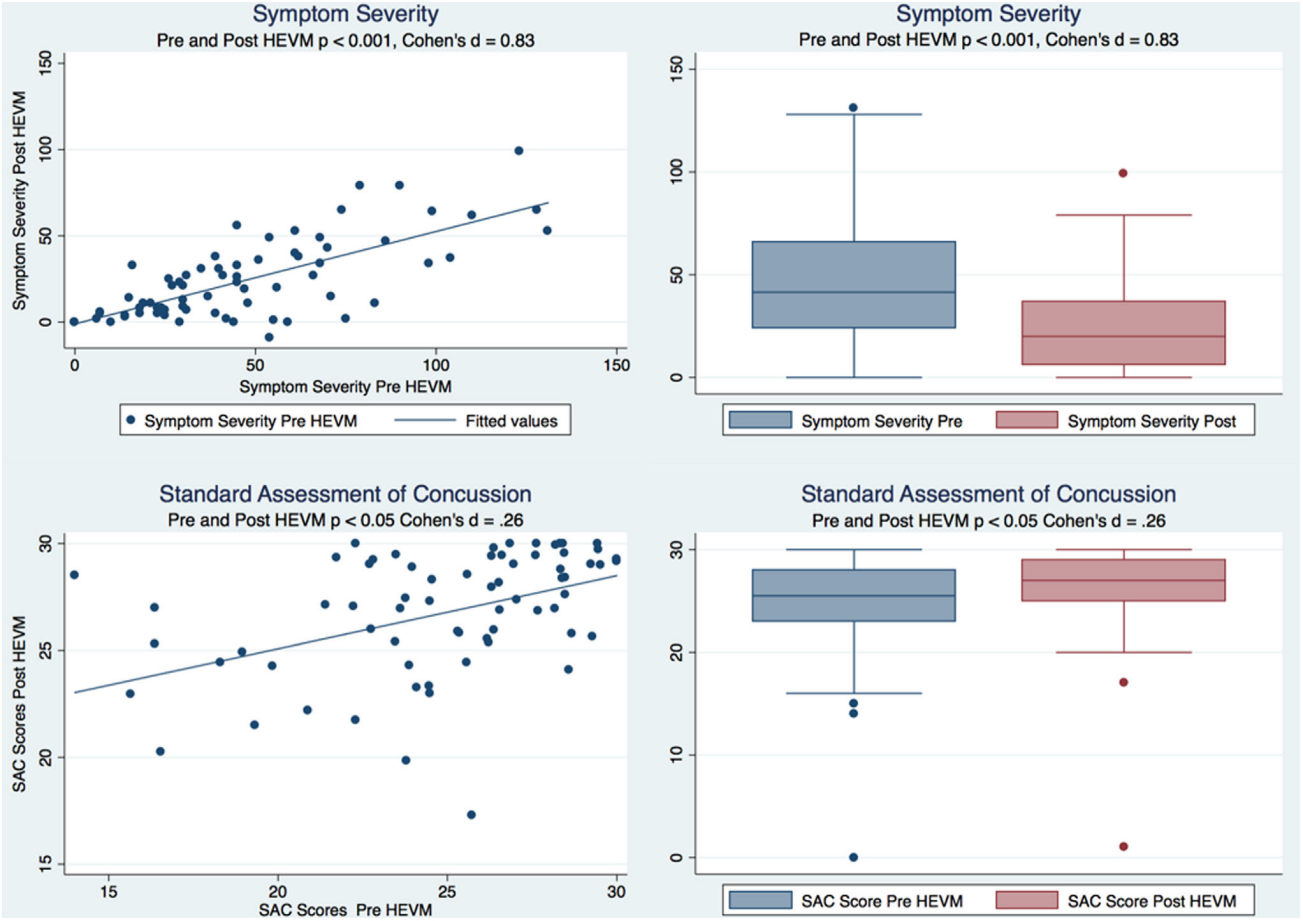
Box plots and regression models of symptom severity and SAC scores pre- and post-HEVM therapy.

A paired *t* test of the symptom severity scores demonstrated a statistically significant decrease in severity scores after HEVM treatment [*t* (69) = 8.8844, *p* = 0.0000] with a large effect size (Cohen’s *d* = 0.83, 95% CI = 0.4879457–1.179151). A Cohen’s *d* is considered to be small if ≤0.2, medium if ≤0.5 and large if ≥0.8. A paired *t* test of the SAC scores demonstrated a statistically improvement in SAC scores after HEVM treatment [*t* (69) = −2.2663, *p* = 0.0266] with a small effect size (Cohen’s *d* = −0.2599813, 95% CI = −0.5922232–0.073194). Figure 1 describes the symptom severity and SAC scores pre- and post HEVM therapy.

**Figure 2:**
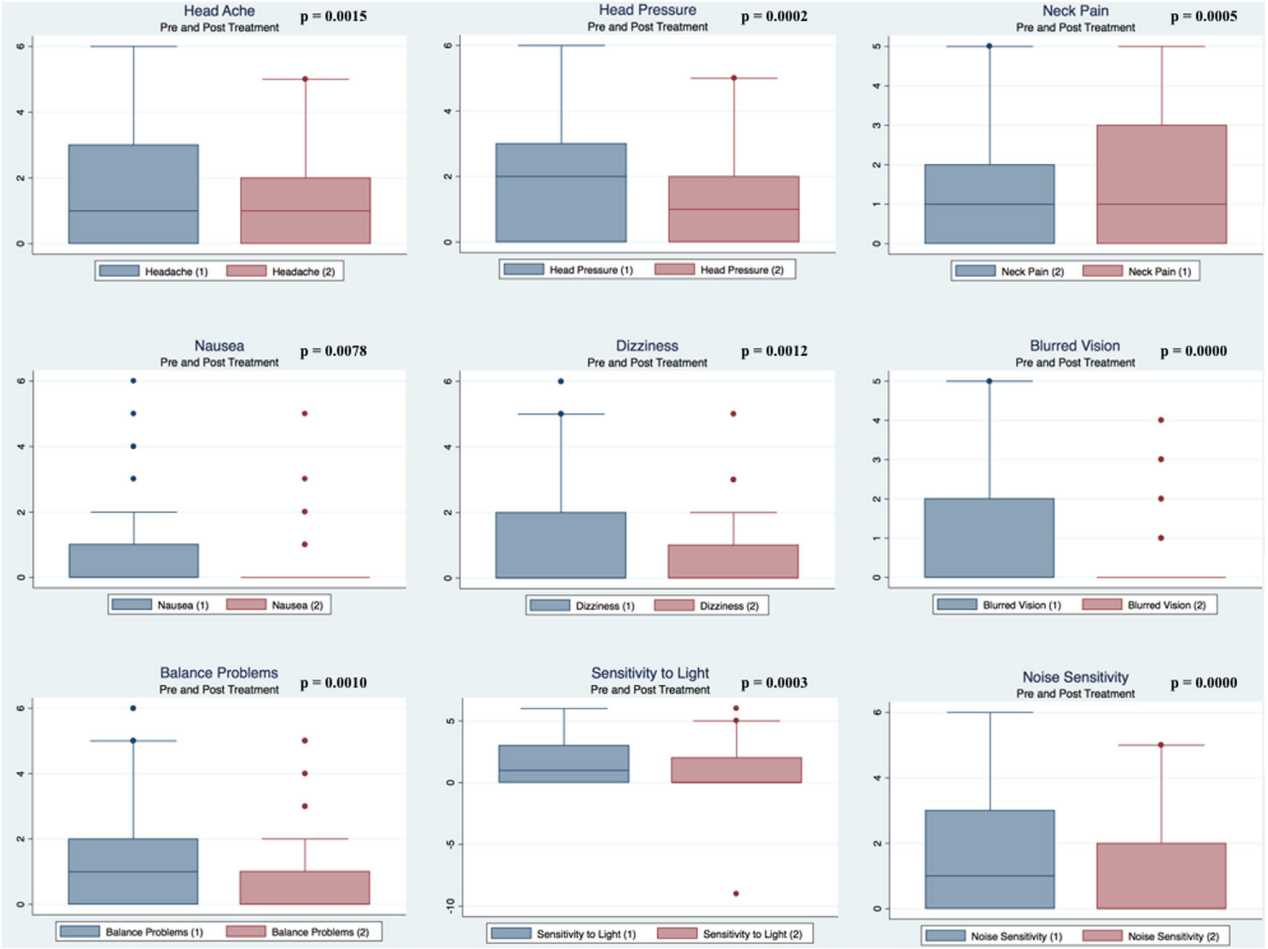
Box plots of C3 Logix variables pre- and post-HEVM therapy.

**Figure 3:**
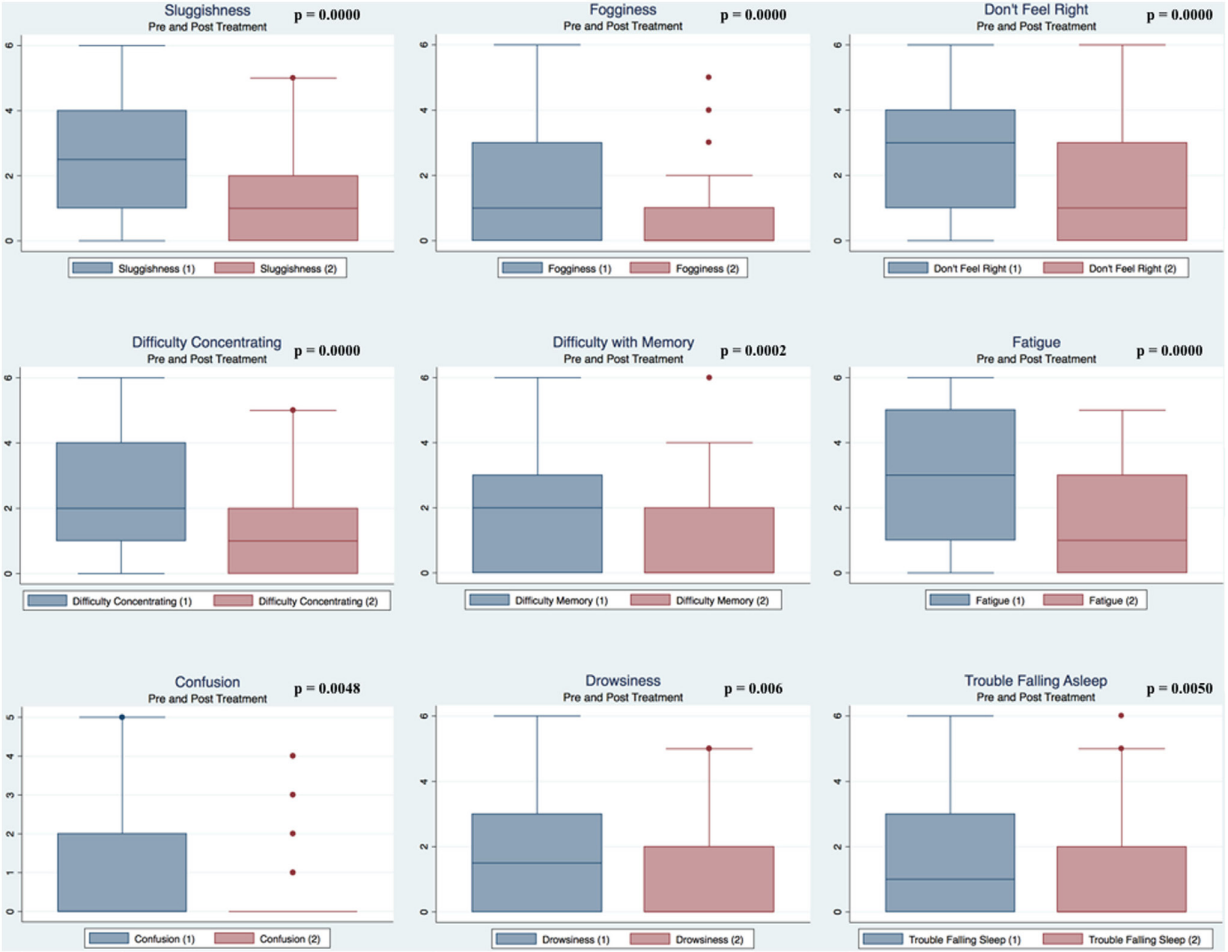
Box plots of C3 Logix variables pre- and post-HEVM therapy.

**Figure 4:**
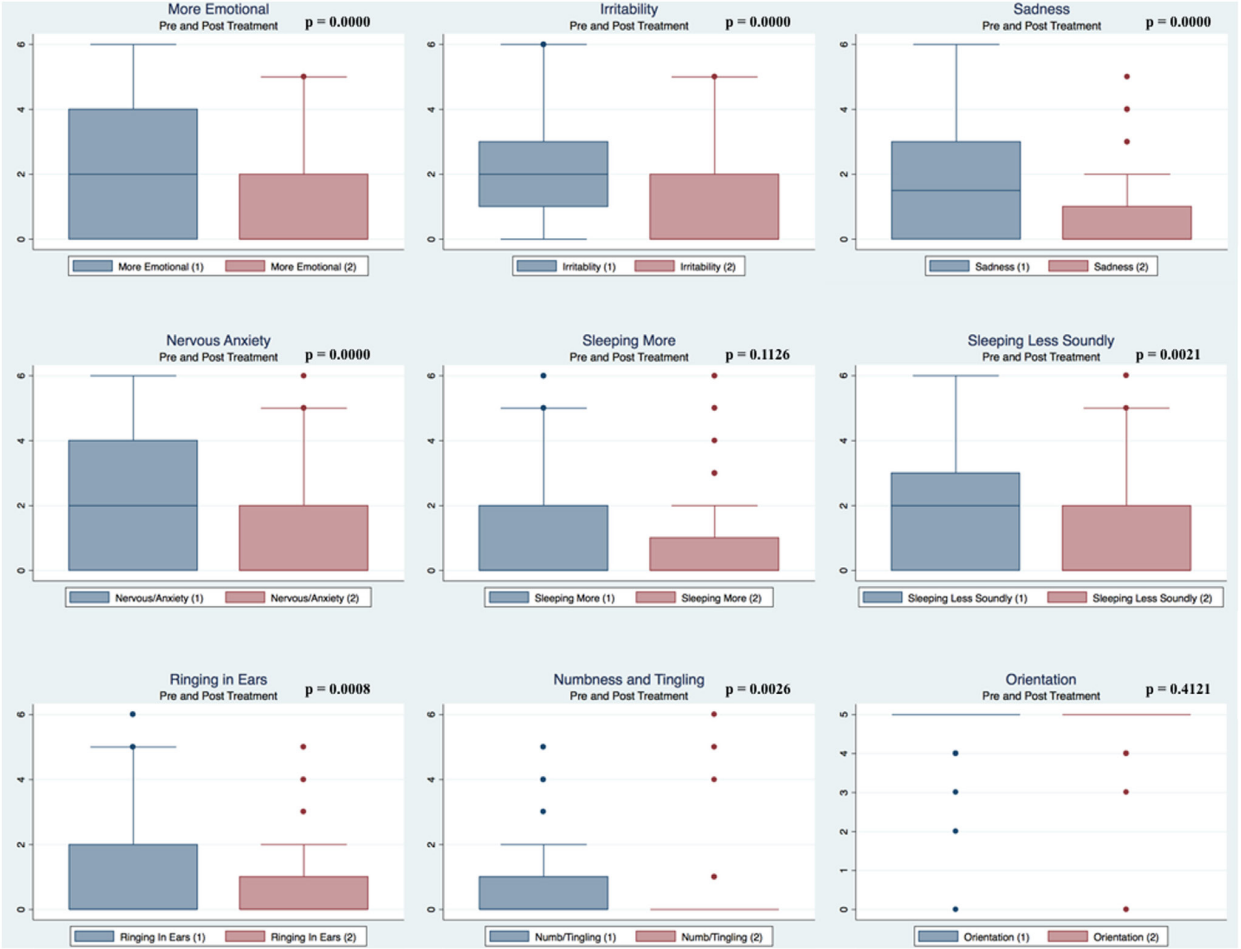
Box plots of C3 Logix variables pre- and post-HEVM therapy.

**Figure 5:**
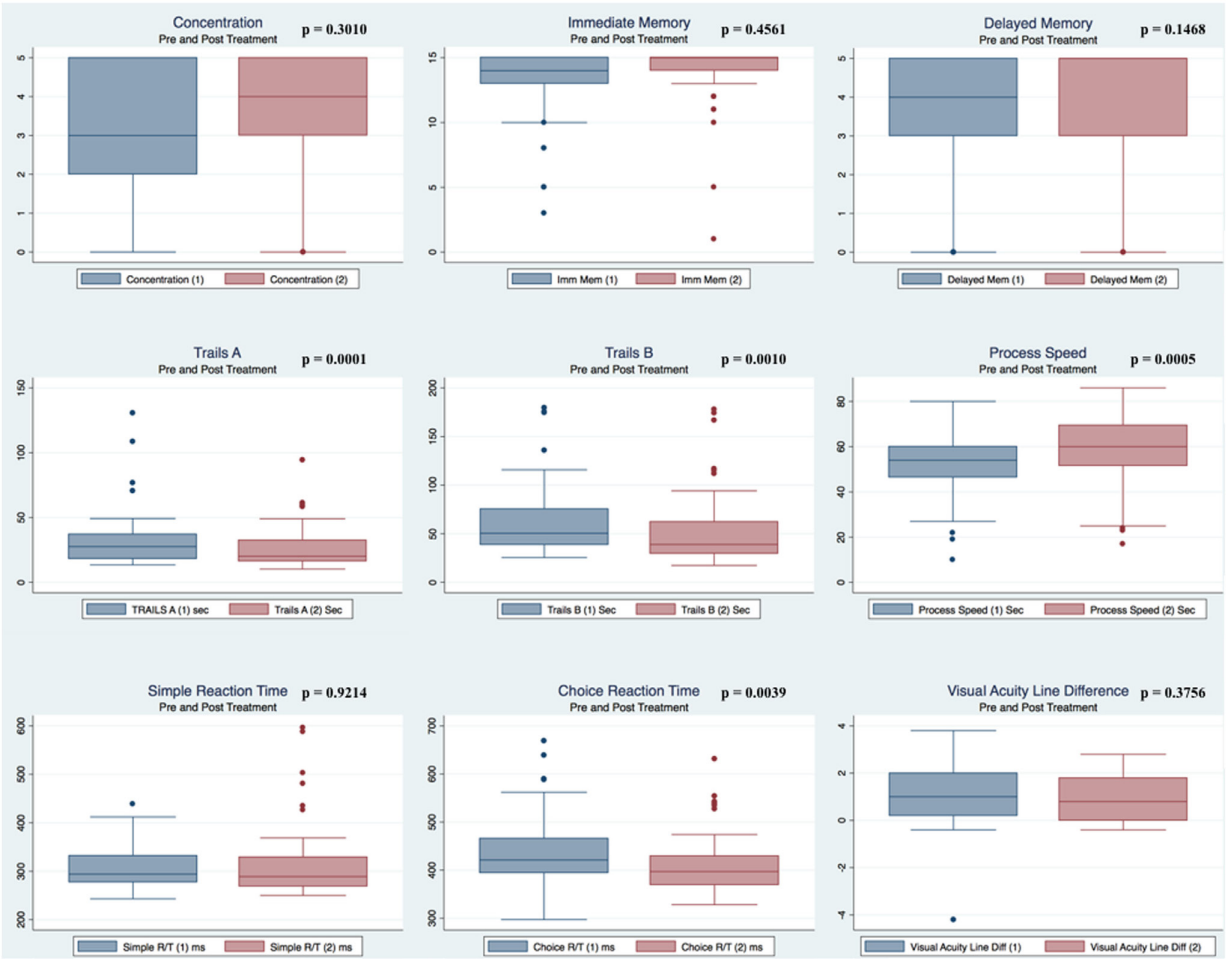
Box plots of C3 Logix variables pre- and post-HEVM therapy.

Pre- and postpaired *t* tests of all C3 Logix variables demonstrated that only 10 of the 40 pre-HEVM variables were not associated with a statistical or substantively significant positive change in outcomes [Sleeping more, Sleeping less, Orientation, Concentration, Immediate Memory, Delayed Memory, Simple reaction time, Visual acuity line difference, Static and Dynamic Visual Acuity (LogMar)]. In all, 75% of the variables tested demonstrated strong statistical significance, with most of these variables demonstrating medium to large substantively significant outcomes. Figures 2–5 describe the changes in C3 Logix Variables Pre and Post HEVM Therapy.

### Multiple Regression Model of Severity Scores Pre-HEVM Treatment

There was a highly significant relationship between longstanding symptom severity and the set of predictors used in the C3 Logix diagnostic platform [*F* (40,29) = 160.17, *p* < 0.0001] before HEVM therapy. The regression model explains 99% of the variance in severity scores (*R*^2^ = 0.9955, adjusted *R*^2^ = 0.9893) and this was an extremely strong relationship. Table 1 describes the statistical and substantive significant changes in Pre and Post C3 Logix Scores after a 5-day program of HEVM in 70 PCS subjects. Of the 40 variables, 18 were statistically significant predictors of the multiple regression model. Table 2 describes the multiple regression model of pretreatment predictors to the pretreatment severity scores.

**Table 1:** Paired *t* tests of pre- and post-C3 Logix scores and their statistical and substantive significance after a 5-day program of HEVM of 70 PCS subjects.

**Variable**	**Mean**	**SD**	**95% CI**	***t***	***p***	**Cohen’s ***d*****
Pre-SAC score	24.44	4.88	23.27923 to 25.6048	−2.27	**0.0300**	−0.2600
Post-SAC score	25.86	5.95	24.43905 to 27.27523			
Presymptom severity	47.14	31.16	39.71206 to 54.57366	8.88	**0.0000**	0.8349
Postsymptom severity	24.23	23.13	18.71255 to 29.74459			
Preheadache	1.77	1.66	1.660901 to 1.375401	3.30	**0.0015**	0.4308
Postheadache	1.03	1.79	0.6028629 to 1.45428			
Prehead pressure	1.87	1.74	1.457596 to 2.285261	3.87	**0.0002**	0.4798
Posthead pressure	1.03	1.78	0.6048028 to 1.45234			
Preneck pain	1.80	1.83	1.363533 to 2.236467	3.64	**0.0005**	0.3819
Postneck pain	1.08	1.91	0.6305019 to 1.540927			
Prenausea	0.70	1.34	0.3794538 to 1.020546	2.74	**0.0078**	0.3876
Postnausea	0.17	1.38	−0.1582953 to 0.5011524			
Predizziness	1.04	1.51	0.6833319 to 1.402382	3.37	**0.0012**	0.4471
Postdizziness	0.37	1.50	0.0148127 to 0.7280445			
Preblurred vision	1.14	1.51	0.7836759 to 1.502038	4.43	**0.0000**	0.5154
Postblurred vision	0.36	1.54	−0.0105405 to 0.7248262			
Prebalance problems	1.33	1.70	0.9231873 to 1.733956	3.42	**0.0010**	0.4361
Postbalance problems	0.58	1.70	0.1787652 to 0.9926633			
Presensitivity to light	1.57	1.81	1.140717 to 2.00214	3.76	**0.0003**	0.3797
Postsensitivity to light	0.87	1.88	0.4231867 to 1.31967			
Prenoise sensitivity	1.86	1.91	1.400949 to 2.313337	4.67	**0.0000**	0.4763
Postnoise sensitivity	0.91	1.93	0.4837823 to 1.401932			
Presluggishness	2.40	1.91	1.9436 to 2.8564	5.08	**0.0000**	0.6781
Postsluggishness	1.10	1.92	0.6422476 to 1.557752			
Prefogginess	1.71	1.83	1.276795 to 2.151776	4.34	**0.0000**	0.5410
Postfogginess	0.73	1.81	0.2971908 to 1.159952			
Pre-don’t feel right	2.9	1.96	2.431572 to 3.368428	5.71	**0.0000**	0.6617
Post-don’t feel right	1.5	2.26	0.9618292 to 2.038171			
Predifficult concentrate	2.5	1.86	2.055768 to 2.944232	5.92	**0.0000**	0.7565
Postdifficult concentrate	1.07	1.91	0.6151701 to 1.527687			
Predifficult memory	1.94	1.81	1.511545 to 2.37417	4.00	**0.0002**	0.5760
Postdifficult memory	0.88	1.86	0.4418663 to 1.329562			
Prefatigue	2.91	1.98	2.441319 to 3.387253	5.56	**0.0000**	0.7137
Postfatigue	1.48	2.02	1.004115 to 1.967313			
Preconfusion	0.98	1.50	0.6283565 to 1.343072	2.91	**0.0048**	0.3894
Postconfusion	0.38	1.58	0.0086114 to 0.7628172			
Predrowsiness	1.77	1.86	1.328271 to 2.214586	3.60	**0.0006**	0.4487
Postdrowsiness	0.92	1.90	0.4759392 to 1.381204			
Pretrouble fall asleep	1.68	2.00	1.207894 to 2.163535	2.90	**0.0050**	0.3574
Posttrouble fall asleep	0.97	1.99	0.4963259 to 1.446531			
Premore emotional	2.1	2.01	1.619416 to 2.580584	4.65	**0.0000**	0.6783
Postmore emotional	0.78	1.85	0.3430746 to 1.228354			
Preirritability	2.16	1.86	1.713123 to 2.601163	5.26	**0.0000**	0.7724
Postirritability	0.74	1.80	0.3138433 to 1.171871			
Presadness	1.81	1.78	1.387955 to 2.240616	4.56	**0.0000**	0.6694
Postsadness	0.63	1.75	0.2102826 to 1.04686			
Prenervous anxiety	2.25	2.07	1.762141 to 2.752145	5.18	**0.0000**	0.6769
Postnervous anxiety	0.88	1.97	0.4148424 to 1.356586			
Presleeping more	1.05	1.74	0.6413944 to 1.472891	1.60	0.1126	0.2250
Postsleeping more	0.64	1.93	0.1817229 to 1.103991			
Presleeping less	1.20	1.96	0.7325403 to 1.66746	1.99	0.0505	0.2508
Postsleeping less	0.71	1.91	0.2583498 to 1.170222			
Presleep less soundly	1.98	2.04	1.497741 to 2.473687	3.19	**0.0021**	0.3665
Postsleep less soundly	1.20	2.24	0.6666739 to 1.733326			
Preringing in ears	1.47	2.01	0.9915803 to 1.951277	3.52	**0.0008**	0.4077
Postringing in ears	0.68	1.84	0.247471 to 1.123958			
Prenumb and tingling	0.87	1.49	0.5153908 to 1.227466	3.12	**0.0026**	0.3918
Postnumb and tingling	0.26	1.63	−0.133679 to 0.6479647			
Preorientation	4.68	0.79	4.497296 to 4.874133	0.82	0.4121	0.1293
Postorientation	4.50	1.87	4.053917 to 4.946083			
Preconcentration	3.38	1.50	3.028818 to 3.742611	−1.04	0.3010	−0.1255
Postconcentration	3.61	2.09	3.114963 to 4.113609			
Preimmediate memory	13.2	2.69	12.55865 to 13.84135	−0.75	0.4561	−0.1001
Postimmediate memory	13.51	3.53	12.67176 to 14.35681			
Predelayed memory	3.48	1.56	3.114225 to 3.857204	−1.47	0.1468	−0.1971
Postdelayed memory	3.84	2.03	3.35803 to 4.327684			
Pretrails A	30.46	20.27	25.62686 to 35.296	4.25	**0.0001**	0.3584
Posttrails A	23.93	15.88	20.14723 to 27.72134			
Pretrails B	60.21	34.95	51.8768 to 68.5432	3.44	**0.0010**	0.3310
Posttrails B	48.45	36.08	39.8464 to 57.0536			
Preprocess speed	51.04	17.19	46.94288 to 55.14284	0.99	**0.0005**	−0.2651
Postprocess speed	55.80	18.65	51.35219 to 60.24781			
Presimple Rx time	304.10	56.45	290.6393 to 317.5607	0.10	0.9214	0.0100
Postsimple Rx time	303.35	87.94	282.387 to 324.3272			
Prechoice Rx time	412.56	125.49	382.6356 to 442.4787	2.99	**0.0039**	0.2534
Postchoice Rx time	380.98	123.69	351.4929 to 410.4785			
Previs acuity line diff	−0.04	3.42	−0.8585596 to 0.7728454	0.89	0.3756	0.0683
Postvis acuity line diff	−0.27	3.26	−1.050304 to 0.5074468			
Prestatic visual LogMar	−1.53	3.25	−2.310766 to 0.7606625	−0.6582	0.5126	−0.0656
Poststatic visual LogMar	−1.33	2.97	−2.039554 to 0.6233027			
Predynamic vis LogMar	−1.47	3.28	−2.25228 to 3.687717	−0.7184	0.4749	−0.0723
Postdynamic vis LogMar	−1.24	3.00	−1.95898 to −0.525585			

*P values < 0.05 are in BOLD. A Cohen’s d is considered to be small if ≤ 0.2, medium if ≤ 0.5 and large if ≥ 0.8*.

**Table 2:** Multiple regression model predictors of presymptom severity scores before HEVM therapy.

Variable	Coef	SE	95% CI	*t*	*p*
Preheadache	0.1249663	0.424072	−0.7423583 to 0.992291	0.29	0.770
Prehead pressure	1.38181	0.5725066	0.2109028 to 2.552718	2.41	**0.022**
Preneck pain	1.048625	0.4136755	0.2025633 to 1.894686	2.53	**0.017**
Prenausea	1.13502	0.4970904	0.1183555 to 2.151684	2.28	**0.030**
Predizziness	1.608892	0.4634325	0.6610656 to 2.556718	3.47	**0.002**
Preblurred vision	0.523634	0.5739241	−0.6501726 to 1.697441	0.91	0.369
Prebalance problems	0.7831329	0.4329659	0.9231873 to 1.733956	1.081	0.081
Presensitivity to light	1.307011	0.4117404	0.4649068 to 2.149114	3.17	**0.004**
Prenoise sensitivity	0.8852479	0.4195145	0.0272445 to 1.743251	2.11	**0.044**
Presluggishness	1.286771	0.5566672	0.1482588 to 2.425283	2.31	**0.028**
Prefogginess	0.7820708	0.5115418	−0.2641497 to 1.828291	1.53	0.137
Pre-don’t feel right	0.6171734	0.4564156	−0.3163014 to 1.550648	1.35	0.187
Predifficult concentrate	0.9412827	0.5122838	−0.1064552 to 1.989021	1.84	0.076
Predifficult memory	1.147174	0.4756962	0.1742659 to 2.120082	2.41	**0.022**
Prefatigue	1.663196	0.5052607	0.6298217 to 2.69657	3.29	**0.003**
Preconfusion	1.249483	0.5761913	0.071039 to 2.427926	2.17	**0.038**
Predrowsiness	0.4915147	0.5273061	−0.5869473 to 1.569977	0.93	0.359
Pretrouble fall asleep	0.1993855	0.4245459	−0.6689085 to 1.067679	0.47	0.642
Premore emotional	1.45038	0.4439555	0.5423892 to 2.358371	3.27	**0.003**
Preirritability	1.606443	0.3952294	0.7981083 to 2.414778	4.06	**0.000**
Presadness	0.4039065	0.4870462	−0.5922148 to 1.400028	0.83	0.414
Prenervous anxiety	0.9107474	0.4125011	0.0670879 to 1.754407	2.21	**0.035**
Presleeping more	0.9503331	0.437544	0.0554551 to 1.845211	2.17	0.038
Presleeping less	1.207889	0.3524688	0.4870091 to 1.928768	3.43	0.002
Presleep less soundly	1.508031	0.3943347	0.7015262 to 2.314536	3.82	**0.001**
Preringing in ears	0.8857425	0.3527799	0.1642265 to 1.607258	2.51	**0.018**
Prenumb and tingling	1.350737	0.5358607	0.2547786 to 2.446695	2.52	**0.017**
Preorientation	0.2857502	1.304419	−2.382087 to 2.953587	0.22	**0.828**
Preconcentration	−0.0339388	0.5734272	−1.206729 to 1.138852	−0.06	0.953
Preimmediate memory	1.078137	0.5163738	0.0220336 to 2.134240	2.09	**0.046**
Predelayed memory	0.2109774	0.6419859	−1.102031 to 1.523986	0.33	0.745
Pretrails A	−0.0444559	0.0652681	−0.1779442 to 0.0890325	−0.68	0.501
Pretrails B	0.0089195	0.0230615	−0.0382467 to 0.0560856	0.39	0.702
Preprocess speed	−0.0541919	0.0593786	−0.1756348 to 0.0672509	−0.91	0.369
Presimple Rx time	304.10	0.0169777	−0.0477724 to 0.0216744	−0.77	0.448
Prechoice Rx time	−0.0020412	0.0062147	−0.0147516 to 0.0106693	−0.33	0.745
Previs acuity line diff	0.1082193	0.2953511	−0.4958415 to 0.7122801	0.37	0.717
Prestatic visual LogMar	−0.2639731	0.3803527	−1.041882 to 0.5139355	−0.69	0.493
Predynamic vis LogMar	0.2609187	0.4228063	−0.6038173 to 1.125655	0.62	0.542
Pre-SAC score	−0.5593229	0.4812354	−1.54356 to 0.4249139	−1.16	0.255
Constant	5.181015	3.440948	−1.856515 to 12.21854	1.51	0.143

*P values < 0.05 are in BOLD*.

**Figure 6:**
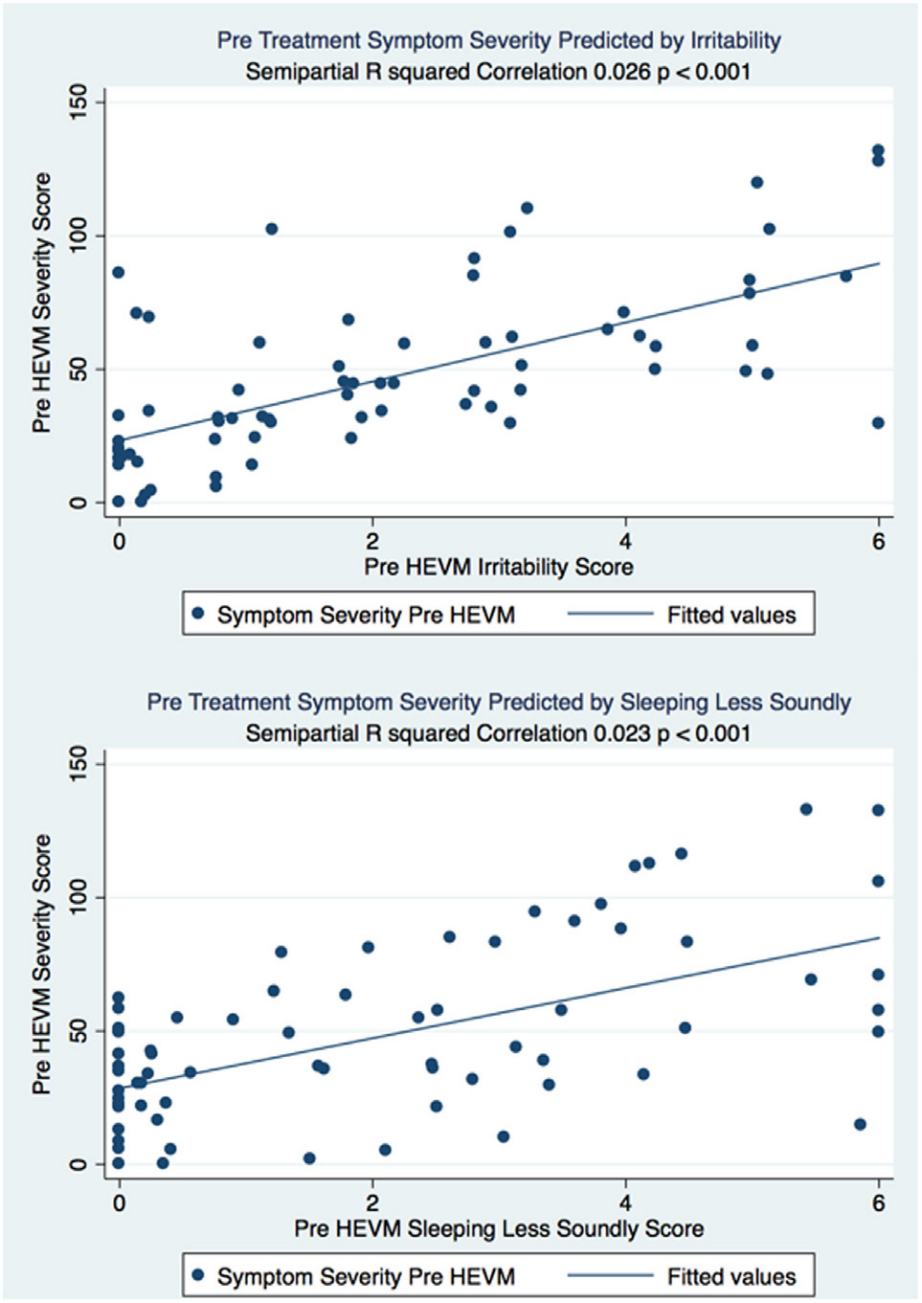
Semipartial R^2^ of the correlations of symptom severity Pre-HEVM treatment with irritability and sleeping less soundly.

The semipartial *R*^2^ of the correlations of symptom severity with each variable demonstrated that 18 of the 40 variables had a statistically significant effect in the prediction of the symptom severity scores. The performance of standard concussions tests did not have a statistically significant predictor effect whereas mental health-associated issues and symptoms did. Irritability and sleeping less soundly were the greatest predictors. Table 3 describes the semi partial *R*^2^ demonstrating how much each variable contributes uniquely to the symptom severity scores before HEVM therapy. Figure 6 represents the semipartial *R*^2^ of the correlations of symptom severity Pre-HEVM treatment with irritability and sleeping less soundly.

**Table 3:** Semipartial *R*^2^ correlations of symptom severity scores before HEVM therapy.

Variable	Semipartial correlations	*p*
Preheadache	0.0000	0.7703
Prehead pressure	0.0010	**0.0223**
Preneck pain	0.0010	**0.0169**
Prenausea	0.0008	**0.0299**
Predizziness	0.0019	**0.0016**
Preblurred vision	0.0001	0.3691
Prebalance problems	0.0005	0.0809
Presensitivity to light	0.0005	0.0809
Prenoise sensitivity	0.0007	**0.0436**
Presluggishness	0.0008	**0.0281**
Prefogginess	0.0004	0.1371
Pre-don’t feel right	0.0003	0.1868
Predifficult concentrate	0.0005	0.0764
Predifficult memory	0.0009	**0.0224**
Prefatigue	0.0017	**0.0026**
Preconfusion	0.0007	**0.0385**
Predrowsiness	0.0001	0.3590
Pretrouble fall asleep	0.0000	0.6421
Premore emotional	0.0017	**0.0028**
Preirritability	0.0026	**0.0003**
Presadness	0.0001	0.4137
Prenervous anxiety	0.0008	**0.0353**
Presleeping more	0.0007	**0.0382**
Presleeping less	0.0018	**0.0018**
Presleep less soundly	0.0023	**0.0006**
Preringing in ears	0.0010	**0.0179**
Prenumb and tingling	0.0010	**0.0175**
Preorientation	0.0000	0.8281
Preconcentration	0.0000	0.9532
Preimmediate memory	0.0007	**0.0457**
Predelayed memory	0.0000	0.7448
Pretrails A	0.0001	0.5012
Pretrails B	0.0000	0.7018
Preprocess speed	0.0001	0.3690
Presimple Rx time	0.0001	0.4483
Prechoice Rx time	0.0000	0.7449
Previs acuity line diff	0.0000	0.7167
Prestatic visual LogMar	0.0001	0.4932
Predynamic vis LogMar	0.0077	0.5420
Pre-SAC score	0.0002	0.2546

*P values < 0.05 are in BOLD*.

### Multiple Regression Model Pre-HEVM Treatment Predictors of Post-HEVM

#### Treatment Symptom Severity

There was a statistically significant relationship between the symptom severity post-HEVM treatment and the initial set of predictors used in the C3 Logix diagnostic platform [*F* (40,29) = 3.07, *p* < 0.0011] before HEVM therapy. The regression model explained 55% of the variance in severity scores (*R*^2^ = 0.8091, adjusted *R*^2^ = 0.5458) and this was a strong relationship. However, only 2 of the 40-pre-HEVM treatment variables (predifficulty with memory *p* = 0.020 and sleeping less soundly *p* = 0.040) were statistically significant predictors of the posttreatment severity score multiple regression model. Table 4 describes the multiple regression model of pretreatment predictors to the posttreatment severity scores.

**Table 4:** Multiple regression model pre-HEVM treatment predictors of post-HEVM symptom severity scores.

Variable	Coef	SE	95% CI	*t*	*p*
Preheadache	−1.511053	2.048943	−5.701612 to 2.679507	−0.74	0.467
Prehead pressure	−2.206008	2.766119	−7.863356 to 3.45134	−0.80	0.432
Preneck pain	2.6348	1.998712	−1.453025 to 6.722624	1.32	0.198
Prenausea	−3.39661	2.401738	−8.308716 to 1.515497	−1.41	0.168
Predizziness	0.8026518	2.239117	−3.776857 to 5.382161	0.36	0.723
Preblurred vision	1.050185	2.772968	−4.621171 to 6.72154	0.38	0.708
Prebalance problems	−1.531246	2.091915	−5.809693 to 2.7472	−0.73	0.470
Presensitivity to light	0.2798448	1.989362	−3.788857 to 4.348547	0.14	0.889
Prenoise sensitivity	1.993531	2.026923	−2.151992 to 6.139054	0.98	0.333
Presluggishness	1.261088	2.689589	−4.23974 to 6.761915	0.47	0.63
Prefogginess	0.1533607	2.471562	−4.901551 to 5.208272	0.06	0.951
Pre-don’t feel right	2.754417	2.205215	−1.755753 to 7.264587	1.25	0.222
Predifficult concentrate	5.06664	2.475147	0.0043966 to 10.12888	2.05	0.050
Predifficult memory	−5.681969	2.29837	−10.38266 to 0.9812736	−2.47	**0.020**
Prefatigue	−0.5405752	2.441214	−5.533419 to 4.452268	−0.22	0.826
Preconfusion	4.509188	2.783922	−1.184571 to 10.20295	1.62	0.116
Predrowsiness	2.107529	2.547728	−3.10316 to 7.318219	0.83	0.415
Pretrouble fall asleep	2.180854	2.051233	−2.014389 to 6.376097	1.06	0.296
Premore emotional	−1.519511	2.145012	−5.906554 to 2.867532	−0.71	0.484
Preirritability	−1.546134	1.909587	−5.451679 to 2.359411	−0.81	0.425
Presadness	2.677565	2.353209	−2.135288 to 7.490417	1.14	0.265
Prenervous anxiety	−0.7281974	1.993037	−4.804417 to 3.348022	−0.37	0.717
Presleeping more	1.6209	2.114035	−2.702787 to 5.944586	0.77	0.449
Presleeping less	−0.0952409	1.702986	−3.578237 to 3.387756	−0.06	0.956
Presleep less soundly	4.099488	1.905265	0.2027842 to 7.996192	2.15	**0.040**
Preringing in ears	−0.9073619	1.704489	−4.393433 to 2.57871	−0.53	0.599
Prenumb and tingling	0.4467434	2.589061	−4.84848 to 5.741967	0.17	0.864
Preorientation	2.882352	6.302423	−10.00755 to 15.77226	0.46	0.651
Preconcentration	−0.5318507	2.770567	−6.198296 to 5.134595	−0.19	0.849
Preimmediate memory	−0.6548606	2.494908	−5.757521 to 4.4478	−0.26	0.795
Predelayed memory	0.666252	3.101815	−5.677671 to 7.010175	0.21	0.831
Pretrails A	−0.0841817	0.3153491	−0.7291431 to 0.5607796	−0.27	0.791
Pretrails B	0.1995216	0.1114239	−0.0283659 to 0.4274091	1.79	0.084
Preprocess speed	−0.1443972	0.2868932	−0.7311597 to 0.4423652	−0.50	0.619
Presimple Rx time	−0.0945081	0.0820295	−0.2622773 to 0.0732612	−1.15	0.259
Prechoice Rx time	0.006105	0.0300268	−0.0553067 to 0.0675167	0.20	0.840
Previs acuity line diff	−0.1821058	1.427016	−3.100682 to 2.73647	−0.13	0.899
Prestatic visual LogMar	0.1238949	0.3803527	−3.634643 to 3.882433	0.07	0.947
Predynamic vis LogMar	1.405874	2.042828	−2.772178 to 5.583926	0.69	0.497
Pre-SAC score	0.8942374	2.325133	−3.861194 to 5.649669	0.38	0.703
Constant	−4.990991	16.62526	−38.99347 to 29.01149	−0.30	0.766

*P values < 0.05 are in BOLD*.

### Multiple Regression Model Post-HEVM Treatment Predictors of Post-HEVM Symptom Severity Scores in 70 PCS Subjects

There was a statistically significant relationship between the symptom severity post-HEVM treatment and the set of predictors used in the C3 Logix diagnostic platform [*F* (40,29) = 47.07, *p* < 0.0001] after HEVM therapy. The regression model explains 98% of the variance in severity scores (*R*^2^ = 0.9848, adjusted *R*^2^ = 0.9639) and this was a strong relationship. However, only 9 of the 40 post-HEVM treatment variables were statistically significant predictors of the posttreatment severity scores multiple regression model. Table 5 describes the multiple regression model of posttreatment predictors to the posttreatment severity scores.

**Table 5:** Multiple regression model post-HEVM treatment predictors of post-HEVM symptom severity scores.

Variable	Coef	SE	95% CI	*t*	*p*
Postheadache	1.193364	1.024362	−0.9016911 to 3.28842	1.16	0.254
Posthead pressure	1.48905	1.235904	−1.038656 to 4.016757	1.2	0.238
Postneck pain	0.7244083	0.6946969	−0.6964063 to 2.145223	1.04	0.306
Postnausea	0.8884091	1.281139	−3.508633 to 1.731815	−0.69	0.494
Postdizziness	−1.086133	1.488765	−4.131 to 1.958734	−0.73	0.472
Postblurred vision	2.345634	1.067646	0.162052 to 4.529215	2.2	**0.036**
Postbalance problems	0.7286127	0.7883909	−0.8838278 to 2.341053	0.92	0.363
Postsensitivity to light	1.456879	0.8027066	−0.1848405 to 3.098598	1.81	0.080
Postnoise sensitivity	0.0123044	0.9700972	−1.996376 to 1.971767	−0.01	0.990
Postsluggishness	1.340684	1.197697	−1.108881 to 3.790249	1.12	0.272
Postfogginess	1.039304	1.516318	−2.061914 to 4.140522	0.69	0.499
Post-don’t feel right	1.714319	0.8155409	0.046351 to 3.382288	2.1	**0.044**
Postdifficult concentrate	0.1665482	1.134714	−2.154202 to 2.487299	0.15	0.884
Postdifficult memory	1.979964	1.107364	−0.2848492 to 4.244777	1.79	0.084
Postfatigue	2.26319	1.082561	0.0491038 to 4.477276	2.09	**0.045**
Postconfusion	2.798798	1.555579	−0.382718 to 5.980314	1.8	0.082
Postdrowsiness	−1.356325	0.9575316	−3.314697 to 0.6020475	−1.42	0.167
Posttrouble fall asleep	0.0396267	0.8199091	−1.716529 to 1.637276	−0.05	0.962
Postmore emotional	0.5458254	1.252254	−3.106971 to 2.015321	−0.44	0.666
Postirritability	0.5313364	0.8569001	−2.283894 to 1.221221	−0.62	0.540
Postsadness	2.582877	1.106073	0.3207035 to 4.84505	2.34	**0.027**
Postnervous anxiety	1.400449	1.297696	−1.253637 to 4.054536	1.08	0.289
Postsleeping more	0.0584207	0.7053558	−1.384194 to 1.501035	0.08	0.935
Postsleeping less	1.040471	0.8733075	−0.7456436 to 2.826585	1.19	0.243
Postsleep less soundly	1.119417	0.9668224	−0.8579567 to 3.096791	1.16	0.256
Postringing in ears	1.053799	0.8976849	−0.7821733 to 2.88977	1.17	0.250
Postnumb and tingling	0.1614592	1.277818	−2.77489 to 2.451971	−0.13	0.900
Postorientation	−5.751606	1.038446	−7.875466 to −3.627745	−5.54	**0.000**
Postconcentration	−8.829704	1.002431	−10.87991 to −6.779502	−8.81	**0.000**
Postimmediate memory	−8.030205	1.01244	−10.10088 to −5.959533	−7.93	**0.000**
Postdelayed memory	−8.172702	1.060049	−10.34075 to −6.004658	−7.71	**0.000**
Posttrails A	0.1179022	0.1257473	−0.3750844 to 0.1392799	−0.94	0.356
Posttrails B	0.0027726	0.0437607	−0.0922733 to 0.086728	−0.06	0.950
Postprocess speed	0.0753283	0.0764841	−0.0810993 to 0.2317558	0.98	0.333
Postsimple Rx time	0.0375889	0.0197743	−0.0028542 to 0.078032	1.9	0.067
Postchoice Rx time	0.0023435	0.0095627	−0.0172144 to 0.0219013	0.25	0.808
Postvis acuity line diff	0.1740374	0.440665	−1.075298 to 0.7272237	−0.39	0.696
Poststatic visual LogMar	0.3802936	0.6598102	−0.9691697 to 1.729757	0.58	0.569
Postdynamic ViLogMar	0.0508954	0.6860482	−1.352231 to 1.454022	0.07	0.941
Post-SAC score	7.934895	0.910908	6.071879 to 9.797911	8.71	**0.000**
Constant	−16.47868	6.524825	−29.82344 to −3.133911	−2.53	0.017

*P values < 0.05 are in BOLD*.

## Discussion

This retrospective study may contribute to an evidence-based treatment for concussions that is reliable and effective but has not been available. All subjects in this study suffered from chronic PCS of greater than 6 months and were refractory to standard medical interventional treatments. They reported a significant decrease in their symptoms and an increase of function after HEVM therapy. HEVM therapy of 5 days duration is associated with statistical and substantive significant decreases of symptom severity associated with chronic PCS. The changes in mental health and physical symptoms after HEVM therapy in PCS patients suggest that this therapy might have applications in other mental health scenarios.

The success of this therapy is associated with activation of the somatic, vestibular, and ocular systems by movements of the head, eye, and body. Movement of the head is associated with otolith stimulation that engages brainstem structures both within and outside of the vestibular nuclear complex, many of which project to the cerebellum (37). These PCS patients all had visual and neurological impairment similar to what is experienced with deficits of vestibular function (38). Their symptoms decreased and performance increased after off-vertical axis rotation (OVAR) that was associated with eye-velocity modulation (39).

Constant velocity OVAR associated with our vestibular therapy provides dynamic linear acceleration stimuli that can stimulate otolith function (40). Otolith-sensitive neurons are activated by the vector of gravity comparative to the head in rotation with encoding of angular velocity resulting in spatiotemporal phenomenology of two dimensions acting as one-dimensional rate sensors (41). When people move their heads they generate reflexive eye movements that represent the motions that PCS patients perceive better than a one-dimensional clinical model (42).

Head–eye vestibular motion appears to produce the integration of neck proprioceptive and vestibular inputs; however, the central integration of sensory activation is different across species (43) making our understanding difficult. For instance, neurons that are responsive to periodic whole body rotation in the alert monkey are located in the caudal parabrachial nucleus, responding to postural aberrancies in locomotion (44). We suggest that during HEVM therapy that reticular neurons take part in the neck tuning of vestibulospinal reflexes (VSRs) by transforming a head-driven sensory input into a body-centered postural response (45).

Three-dimensional space whole body rotation of our patients evokes both static and dynamic vestibular activation that produce postural adaptation depending upon the frequency of activation and the position of the limbs, trunk and head (46). We understand that the VSR gain in whole animal rotation results in changes of foot posture during tilt force (47) and limb muscle activity is modified by rotational activation of VSRs that affect the integrity of posture and balance (48). We have observed increased postural reflexes and orientation of our patients in this study at rest and in movement (49) that is associated with a central integration of trunk in space coordinates as a consequence of head position (49).

Head–eye vestibular motion therapy is directed to change both the phase difference and gain ratio of the neck to the vestibular response affecting postural responses by utilizing vestibular and reticular targets (50). The conscious perception of passive horizontal rotations of the trunk, the head, or both depends on the interaction of canal and neck afferents associated with postural reflexes and neuronal responses (51). HEVM therapy stimulates the vestibular system and this stimulation appears to decrease the symptom severity of chronic PCS with non-vestibular symptom and functional changes. The generation of both voluntary and reflexive orientating head movements during HEVM therapy is mediated by complex pathways involving the cerebral cortex and superior colliculus while stabilization is thought to be mediated by simple short-loop pathways that generate vestibulocollic (VCR) and cervicocollic (CCR) reflexes (52). The VCR and CCR attempt to stabilize head position in space during whole body movements and are subserved by relatively direct, as well as indirect pathways linking vestibular nerve activity to cervical motor neurons (53, 54).

Head stability is important during HEVM therapy with human balance corrections and the VCR modulating mechanically induced instability of the head and neck (55). The majority of our PCS patients complain of stability problems when moving their heads and we know that the short-latency VCR is not suppressed by active head turns (56). Neck muscles are activated by the VCR and resist the direction of movement of the head rotation with functional and rehabilitative consequences (57) if the VCR is intact. For instance, we know that transient passive head rotations in PD patients are followed by an initial rapid rise in resistive torque representing reflexive head stabilization that normal subjects are able to suppress (58). PD patients have gait instability and often have an absent VCR (59), prompting us to consider a common etiology in PCS patients that share much of the same physical symptoms as those we observed in our subjects.

Head–eye vestibular motion utilizes a combination of active trunk mechanics and vestibular integration in order to coordinate head and trunk motion (60). It appears that HEVM activates the CCR and VCR acting together as well as individually to prevent oscillation of the head when the body is still (61). The stabilization of head motion through HEVM therapy seems to contribute to increasing function and decreasing the symptoms of PCS even though many of those symptoms are not traditionally classified as vestibular.

Head–eye vestibular motion induces angular velocities of the head and trunk in yaw and pitch with greater phase shifts observed in pitch over yaw rotation (62) with HEVM treatments activating proprioceptors in the neck that integrate centrally in the vestibular system (63). When our patients move their heads they activate the VCR as well as the semicircular canals and otoliths of the vestibular system contributing to stabilization of the head in space (64). We expect the evocation of a broad frequency of response to the central integration of semicircular canal and otolitic activation (65) thus promoting linear acceleration detection generated by both static tilt of the head in reference to gravity and dynamic linear translation (66, 67).

The convergence of canal and otolith inputs contribute mainly to VSRs by sending inputs to the neck and other muscles during head inclination (68) but also activate brain structures involved in PCS. A multisensory vestibular-cortical network involving the middle and superior temporal gyri, posterior insular cortex, and the inferior parietal cortex is activated bilaterally by saccular responses to the VCR (69). There are differences between responses to vertical and horizontal rotations (70) and we rotate subjects in combined planes during HEVM therapy. The sensory signals from the semicircular canals in constant-velocity chair rotations undergoes neural processing to compute the percept of self-motion (71) an important contribution to human stabilization. Rotation of the head on the trunk induces the transformation of vestibular signals from head position to trunk-in-space functionality in the vestibular nuclei (49). We expose our PCS patients to head positions that are dependent upon adaptation to body motions and adaptation to head movements performed during fast rotation specific to the particular plane of the head movement (72). Slow and fast walking evokes head pitch movements by the angular VCR and the linear VCR, respectively (73), when our PCS patient’s symptoms decrease and their activities increase.

This continued stimulation would appear to be salubrious. The consequence of head accelerations in HEVM therapy may be partly accomplished by VSR and vestibulo-oculospinal (VOS) convergent neurons involving the oculomotor complex and spinal cord; vestibulo-ocular, vestibulospinal, VOS, and vestibular neurons (74).

It is easier to activate central structures by HEVM movements in the pitch nose down vector and by roll and yaw away from the side of the muscle activated (75). HEVM therapy activates a combination of planes that excite the neuronal pool maximally dependent upon the vector of movement (76).

It is likely that activation of reticulospinal fibers, with their subsequent motor consequences, are significant contributors of the neural substrate of the VCR (77) and are central to our therapy. Reticulospinal fibers make an important contribution to the horizontal VCR and in response to stimuli in vertical planes, the pontomedullary reticulospinal fibers depend on convergence of inputs within the neck with otolith reflexes (78). Natural stimulation of the labyrinth of decerebrate cats in vertical planes evokes responses of pontomedullary reticulospinal neurons, the largest fraction of which project to the lumbar cord, playing a role in gravity-dependent postural reflexes of neck and limbs (79). We attempt to maximize this activation by our combination of complex movements of the head, eyes and extremities. The effectiveness of vestibulospinal and reticulospinal fibers can be modified by spontaneous activity of neurons in the C3 ventral horn subsequent to sinusoidal vestibular stimulation of decerebrate paralyzed cats in multiple vertical planes (80).

All cerebellar patients demonstrate impaired otolith-ocular responses and may demonstrate severe vestibular deficits (81) and problems with balance similar to those demonstrated by our PCS patients. The anterior semicircular canal pathways become more sensitive than posterior semicircular canal pathways in cerebellar disease due to probable disinhibition of the flocculus/paraflocculus resulting in central changes of second order neurons in vestibular nuclei (82). We know that sustained centrifugation decreases gravitational modulation, reflecting a shift toward a more body centered frame of reference (83) and this is what we have observed.

## Conclusion

Head–eye vestibular motion therapy of 5 days duration is associated with statistical and substantive significant decreases of symptom severity associated with chronic PCS. The changes in mental health and physical symptoms after HEVM therapy in PCS patients suggest that this therapy might have applications in other mental health scenarios. Many of the individual C3 Logix pretreatment variables provide good predictability of the total severity scores associated with PCS and good outcome measurements of the success or lack of success of treatments. Irritability and sleep disorders were the greatest predictors of the severity of total symptom scores. C3 Logix pretreatment variable scores do not seem to be predictors of the outcomes of HEVM treatment with the exception of PCS patients with memory difficulties and sound sleeping difficulty. The treatment outcomes are dependent upon receiving the treatment and not the subjective or objective findings before the treatment. All subjects in this study had severe debilitating symptoms that lasted longer than 6 months. A 5-day intensive HEVM therapy scenario with demonstrable success is an effective modality that might be considered in chronic treatment refractory PCS. The C3 Logix concussion system has provided an easy and accurate method of quantifying PCS subject function and disability.

### Strengths and Limitations

The use of C3 Logix has facilitated the collection and comparison of outcomes in this study. Many of the C3 Logix are subjective and may be associated with reporting error that can limit interpretation. This is a retrospective review and no control group has been included in this study. These are major limitations and further investigations with prospective designs including a randomized controlled study are necessary to further our understanding. We have addressed changes measured using the C3 Logix platfrom that we have not been able to measure directly. Specifically we have not measured the activity of specific neuronal populations and their integrative activity with the vestibular ocular system. This is a major limitation of this investigation and one that should be embraced in the follow up studies that we are planning. The inclusion of functional imaging and exacting injury quantification will assist us in understanding the phenomenon of success we have observed in our investigation. We know that HEVM therapy is beneficial but have not compared it to other physical exercises or therapies, except by the history of the patients who have reported failure with all therapies, including quality vestibular and physical rehabilitation at competent facilities. Such comparative studies will allow us to understand the consequences of HEVM therapy better.

## Presentations

This research was presented as a Poster at the 12th World Congress on Brain Injury. New Orleans, LA, United States, March 29–April 1, 2017.

## Ethics Statement

This retrospective records review was carried out in accordance with the recommendations of the Carrick Institute Institutional Review Board with written informed consent from all subjects. All subjects gave written informed consent in accordance with the Declaration of Helsinki. The protocol was approved by the Carrick Institute Institutional Review Board (HHS #: IRB00006615 FWA: 00022305).

## Author Contributions

FC, MA, GP, EO, AH, RZ, and JC contributed to study conception and design, acquisition of data, analysis and interpretation of data, drafting of manuscript, and critical revision.

## Conflict of Interest Statement

The authors declare that the research was conducted in the absence of any commercial or financial relationships that could be construed as a potential conflict of interest.
